# Antibiofilm Activity of *Combretum micranthum* G. Don Catechin–Sugar Phytocomplex on *Pseudomonas aeruginosa*

**DOI:** 10.3390/molecules29092091

**Published:** 2024-05-01

**Authors:** Viviana Teresa Orlandi, Fabrizio Bolognese, Luca Chiodaroli, Ilaria Armenia, Enrico Caruso, Miryam Chiara Malacarne

**Affiliations:** Department of Biotechnology and Life Sciences (DBSV), University of Insubria, Via J.H. Dunant 3, 21100 Varese, Italy; fabrizio.bolognese@uninsubria.it (F.B.); luca.chiodaroli.83@gmail.com (L.C.); ilaria.armenia@uninsubria.it (I.A.); enrico.caruso@uninsubria.it (E.C.); mc.malacarne1@uninsubria.it (M.C.M.)

**Keywords:** *Pseudomonas aeruginosa*, biofilm, antimicrobial treatment, *Combretum micranthum* G. Don, phytocomplex

## Abstract

Clinicians often have to face infections caused by microorganisms that are difficult to eradicate due to their resistance and/or tolerance to antimicrobials. Among these pathogens, *Pseudomonas aeruginosa* causes chronic infections due to its ability to form biofilms on medical devices, skin wounds, ulcers and the lungs of patients with Cystic Fibrosis. In this scenario, the plant world represents an important reservoir of natural compounds with antimicrobial and/or antibiofilm properties. In this study, an extract from the leaves of *Combretum micranthum* G. Don, named Cm4-p, which was previously investigated for its antimicrobial activities, was assayed for its capacity to inhibit biofilm formation and/or to eradicate formed biofilms. The model strain *P. aeruginosa* PAO1 and its isogenic biofilm hyperproducer derivative B13 were treated with Cm4-p. Preliminary IR, UV-vis, NMR, and mass spectrometry analyses showed that the extract was mainly composed of catechins bearing different sugar moieties. The phytocomplex (3 g/L) inhibited the biofilm formation of both the PAO1 and B13 strains in a significant manner. In light of the obtained results, Cm4-p deserves deeper investigations of its potential in the antimicrobial field.

## 1. Introduction

*Pseudomonas aeruginosa* is an opportunistic pathogen capable of causing infections in immunocompromised and hospitalized patients [1]. The worsening of their health may be attributed to the bacterial production of a wide spectrum of virulence factors and to the low efficacy of antibiotics and disinfectants [2]. Furthermore, *P. aeruginosa* cells adhering to medical devices such as prosthesis and catheters or human tissues form biofilms, thus favoring the onset of chronic infections [3,4]. Biofilms are multicellular highly organized communities encased in an extracellular matrix of glycosidic nature, incorporating extracellular proteins and eDNA [5]. The matrix, in combination with persister cells, contributes to the failure of traditional antimicrobials [5,6]. Bacteria inside the biofilm have a decreased growth rate, and related multi-omic analyses compared to those of the planktonic cells confirm a metabolic adaptation [6].

Since in the clinical field, bacterial biofilms are an important concern to face, new anti-biofilm strategies need to be developed. Researchers proceed in investigating new materials and coatings that prevent bacterial adhesion and in quenching quorum sensing (QS) mechanisms that drive biofilm formation. In addition, new compounds that are able to break, detach and possibly eradicate mature biofilms are tested [7]. At present, biofilm disruption and/or corrosion can be obtained by employing metal chelators, such as EGTA (Ethylene Glycol Tetraacetic Acid) and EDTA (Ethylene DiamineTetraacetic Acid) [8]; Sodium Dodecyl Sulfate (SDS) as a surfactant [9]; D-aminoacids [10]; or new photoactivated antimicrobials [11].

Indeed, a new promising source of medical compounds is offered by vegetables which are often used as antimicrobials in traditional medicine. For example, in West Africa, to fight against many health diseases caused by microbial pathogens, local populations use medicinal plants [12,13]. They contain different structural classes of molecules, such as alkaloids, acetylenes, coumarins, flavonoids and isoflavonoids, iridoids, lignans, macrolides, phenols, polypeptides, quinones, steroidal saponins, terpenoids and xanthones [14]. 

The *Combretaceae* plant family is one of the most widely used and appreciated resources for ethnomedical purposes: it comprises about 20 genera and 600 species [15]. It includes forest trees, sometimes exceeding the height of 50 m, dwarf shrubs with subterranean rhizomes and short aerial shoots. In ethnomedicine, many parts of *Combretum* are used, including the leaves, roots, barks or even fruits [16,17]. Since preparations from different species of *Combretum* are frequently employed to treat microbial infections, many studies explored the in vitro antibacterial and antifungal effects of *Combreataceae* extracts [18,19]. Some extracts showed antimicrobial activities when prepared as water decoctions and infusions, while only alcoholic and acetone extracts were found active for some other microorganisms [20]. Combretastatins are bibenzylic molecules that account for the most important antibacterial activity found in *Combretum* spp., while other interesting substances are phenanthrenes, acidic tetra- and penta- cyclic triterpenes, triterpenoids, flavonoids, ellagitannins and saponins [21].

In the present study, we investigated the anti-biofilm potential of an ethanolic extract from the leaves of *Combretum micranthum* G. Don. The extract, called Cm4-p, was obtained through a modification of the extraction protocol developed in a previous work in which its activity was also tested on various bacterial strains [22]. The model microorganism *P. aeruginosa* PAO1 was chosen as a biofilm former both in static and dynamic conditions.

## 2. Results and Discussion

The use of natural products as antimicrobials is an important field that deserves attention. Ethanolic extract from *Combretum micranthum* G. Don leaves (Cm4-p) showed promising antimicrobial activity against both Gram-negative and Gram-positive pathogens [22]. This extract could be obtained quite easily and in good yields (about 1.5 g/100 g leaves), and its antibacterial activity was independently reproducible from the leaf batch and/or collection period [22]. Therefore, it can be considered a good candidate for practical applications thanks to the widespread abundance of the plant. In this work, we investigate the potential use of Cm4-p as an anti-biofilm compound.

### 2.1. Chemical Analysis of Cm4-p Extract

**Figure 1:**
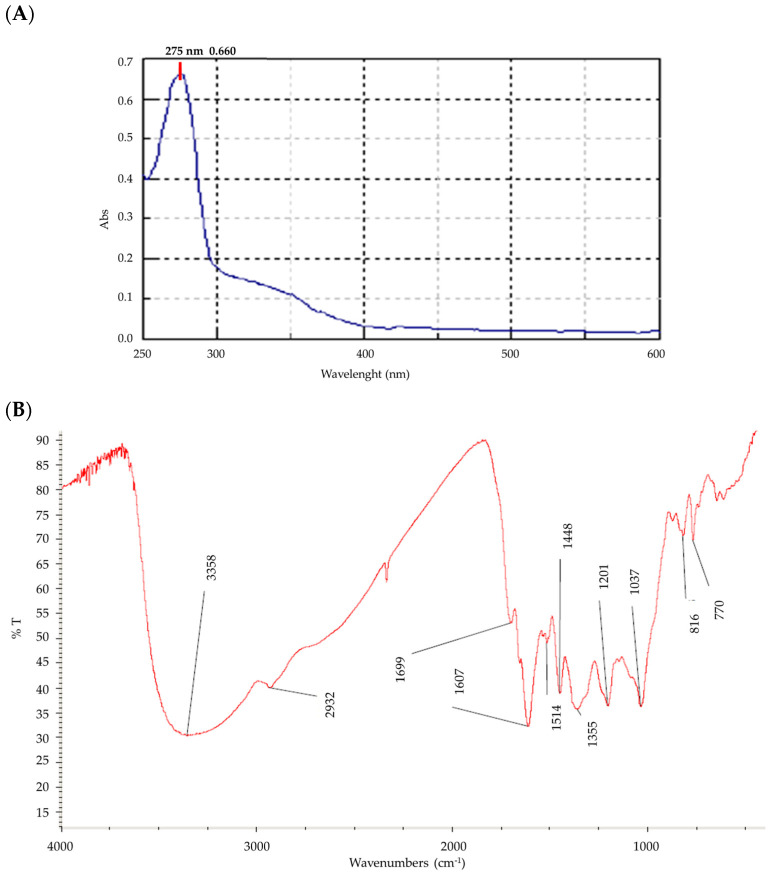
(**A**) UV-Vis spectrum in water of Cm4-p. (**B**) Infrared spectrum of Cm4-p dispersed in KBr.

Preliminary approaches to determine Cm4-p’s chemical composition are described herein. According to a UV-Visible analysis, Cm4-p showed one absorbance peak at 276 nm with a shoulder at 318 nm, a characteristic signal of π-π* transitions of aromatic compounds (Figure 1A). Infrared spectroscopy showed the presence of an intense signal in the range of 3400–3300 cm^−1^, which is indicative of the presence of hydroxyl groups (Figure 1B). As a consequence, a polyphenolic structure of the flavonoid family might be hypothesized for this compound.

**Figure 2:**
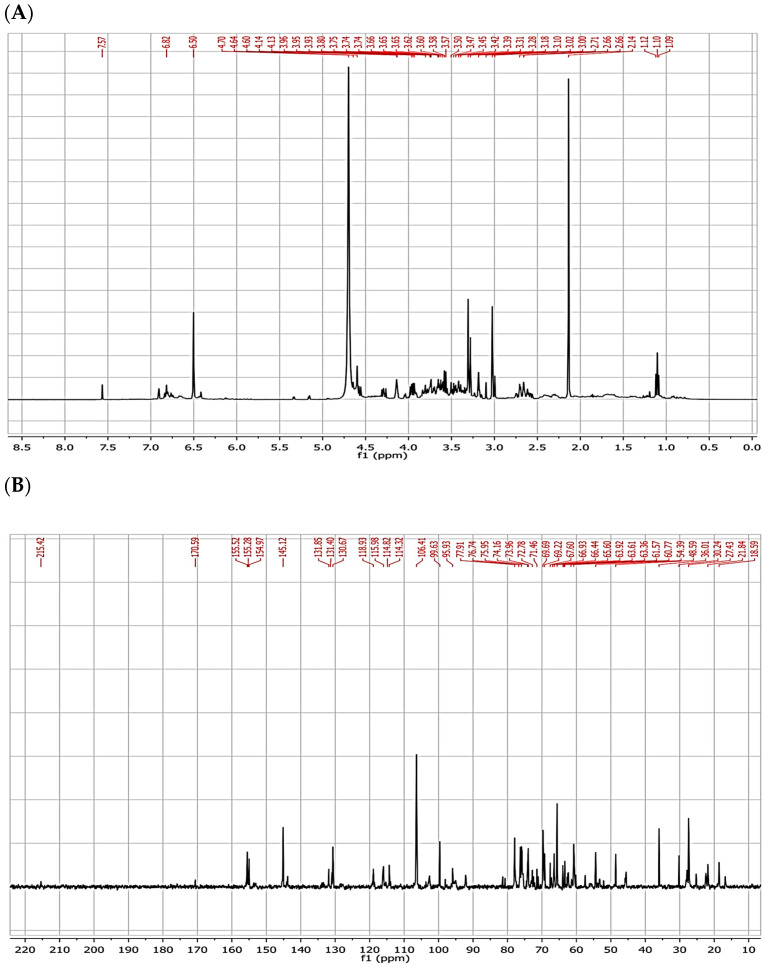
(**A**) ^1^H and (**B**) ^13^C NMR spectra of Cm4-p in D_2_O.

In accordance, in the ^1^H-NMR spectrum (Figure 2A) of Cm4-p, a few aromatic hydrogens and a low number of aliphatic ones could be identified, whereas an abundant presence of hydrogens on carbons bearing heteroatom (3.0–4.5 ppm) was observed. ^13^C-NMR (Figure 2B) showed the absence of carbonyls and the presence of a few sp^2^ hybridized carbons (then few aromatic rings) along with the presence of a small number of aliphatic sp^3^ carbon atoms. As expected from the data obtained with ^1^H-NMR, several sp^3^ hybridized carbons were found in the range of 50–80 ppm (i.e., carbon atoms close to heteroatoms), particularly near oxygen (over 60 ppm) and nitrogen (50–60 ppm) atoms. These spectra clearly indicate the presence of a glycoside structure composed of more than one monosaccharide unit and a glycone part.

**Figure 3:**
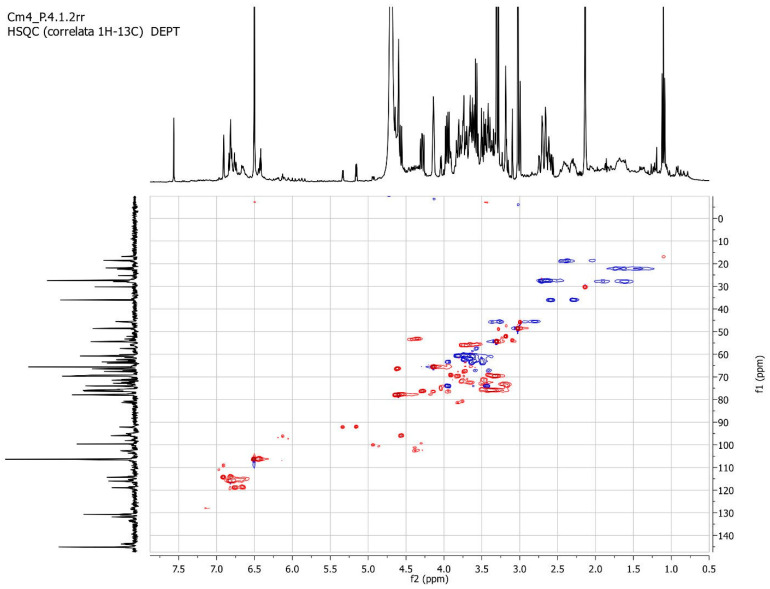
NMR heterocorrelate ^1^H and ^13^C DEPT of Cm4-p.

The heteronuclear single quantum correlation (HSQC) experiment (Figure 3) indicates the presence of two diastereotopic hydrogens upon a single sp^3^ carbon (37 ppm), thus indicating the presence of an adjacent stereogenic center.

**Figure 4:**
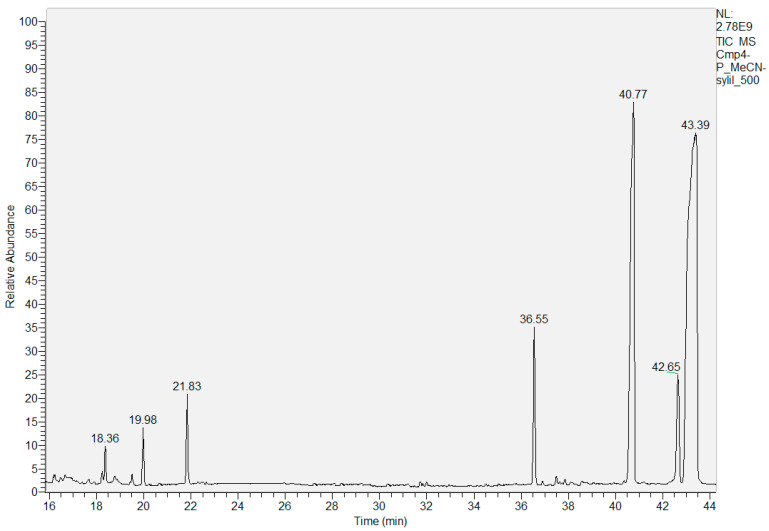
GC-MS analyses of TMS derivative of Cm4-p.

The trimethylsilyl (TMS) derivative of Cm4-p was synthesized for GC/MS analyses. The structures of the TMS derivatives were identified by comparison with the mass spectra of the National Institute of Standards and Technology (NIST) Library, suggesting the presence of some glycosides and an unidentified compound. The following molecules were recognized: D-fructose at 18.36 min, D-glucose at 19.98, one not identified glycoside at 21.83 min (low matching with the structure reported in the NIST Library), a D-turanose at 36.55 min, a catechin at 40.77 min and a 2,6-dihydroxybenzoic acid at 42.65 (Figure 4).

In addition, a compound at 43.39 min, with a molecular peak of *m*/*z* 648, was not identified. All of the analyses support the hypothesis that in Cm4-p, a phytocomplex made up of a catechin linked to a composite glyosidic structure is present.

### 2.2. Effect of Cm4-p on P. aeruginosa Biofilms

**Figure 5:**
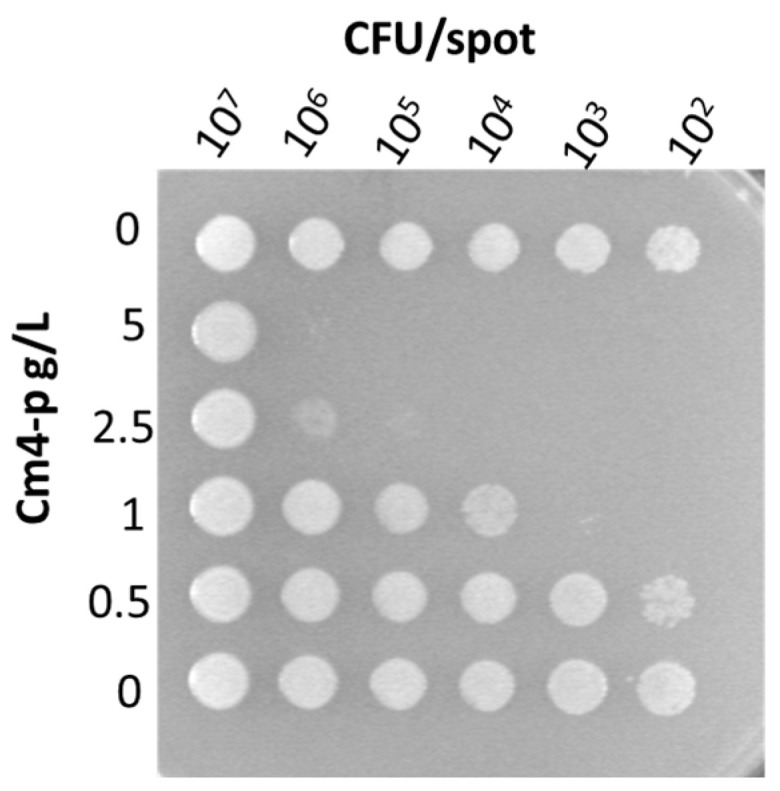
Spot test. Samples of *P. aeruginosa* PAO1 were inoculated at decreasing densities (from 10^7^ up 10^2^ CFU/spot) and treated with increasing concentrations of Cm4-p. Picture is representative of three independent experiments.

Previous investigations highlighted the antimicrobial activity of Cm4-p extract against Gram-positive species, such as *Staphylococcus aureus*, *Staphylococcus xylosus* and *Clostridium difficile*. Interesting results were also obtained with Gram-negative *Escherichia coli* and *P. aeruginosa* [22]. In this study, *P. aeruginosa* was chosen as a model microorganism for two main reasons: it is an opportunistic pathogen that can be isolated from soil, vegetables, plants and water and can cause infections in immunocompromised and defeated patients [23]. Figure 5 shows the representative results of a spot test of *P. aeruginosa* PAO1 serial dilutions challenged with increasing concentrations of Cm4-p. The killing effect was dose-dependent: at the highest extract concentrations (5 and 2.5 g/L), growth inhibition was clearly more efficient than that observed at the lowest ones (0.5 and 1 g/L, respectively). Furthermore, the activity was cell-concentration-dependent, as the compound was not active at the highest cell amount (10^7^ CFU/spot). 

Among bacterial species, *P. aeruginosa* is one of the most tolerant to antimicrobial treatments thanks to its intrinsic resistance mechanisms to antibiotics, the wide arsenal of virulence factors and its ability to form biofilms on inert and living surfaces. Indeed, biofilm formation represents the main survival strategy of bacteria to persist in natural environments [24]. In this scenario, we assayed the potential of Cm4-p as an anti-biofilm compound.

To this aim, microbiological tests were performed on *P. aeruginosa* PAO1 and its isogenic derivative B13, which was previously identified as a biofilm hyperproducer [25]. This transposon mutant, isolated in our laboratory, is characterized by the interruption of the *retS* gene by a gentamycin resistance cassette. *RetS* codifies the regulator of exopolysaccharide and the type III secretion system, which is known to influence biofilm formation [26,27].

**Figure 6:**
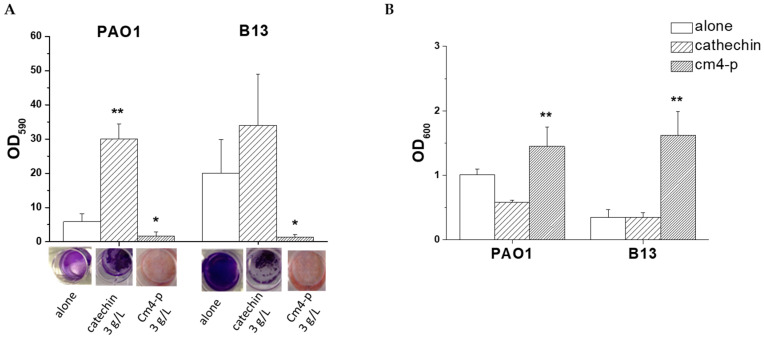
(**A**) Effects of Cm4-p (3 g/L) and catechin (3 g/L) on *Pseudomonas aeruginosa* PAO1 and B13 biofilm formation upon 24 h treatment in static conditions. Adherent biomass after crystal violet staining (OD_590_) and representative images of biofilms after crystal violet staining. (**B**) Planktonic biomass (OD_600_) upon different treatments. Data are means of three independent experiments ± SD. ** *p* < 0.01; * *p* < 0.05.

As shown in Figure 6A, the 24 h old untreated biofilm of *P. aeruginosa* B13 was characterized by an adherent biomass (OD_590_ = 24.46 ± 5.23) 3.5-fold higher than that of wild-type *P. aeruginosa* PAO1 (OD_590_ = 6.90 ± 1.43). On the other hand, the planktonic biomass of the B13 strain (OD_600_ = 0.29 ± 0.07) was lower than that of PAO1 (OD_600_ = 1.01 ± 0.09) (Figure 6B). The data show that, under the tested conditions, B13 (BFI = 93.93 ± 25.20) was a better biofilm former than PAO1 (BFI = 4.99 ± 2.81).

#### 2.2.1. Effect of Cm4-p on *P. aeruginosa* Biofilm Formation

**Figure 7:**
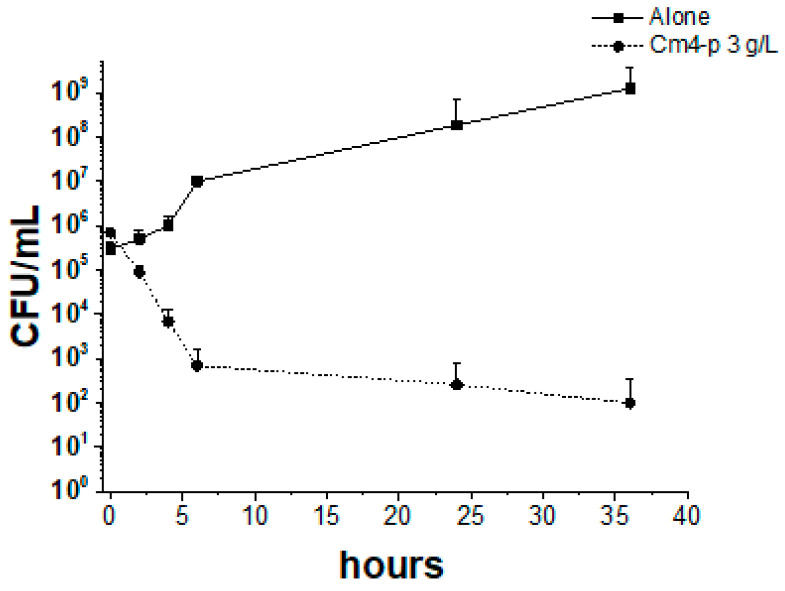
Time-killing assay of Cm4-p (3 g/L) on *Pseudomonas aeruginosa* PAO1. Control cultures are reported with solid lines, whereas treated cultures are represented with dashed lines. Data are means of three independent experiments ± SD.

To evaluate the potential of Cm4-p on biofilm formation, the phytocomplex was administered to PAO1 and B13 suspensions. Bacteria were treated with 3 g/L of Cm4-p, a concentration ~2.5-fold higher than the MIC value (1.25 g/L) previously obtained by authors following the official CLSI protocol on samples at 10^5^ CFU/mL [22]. According to the experimental results of the spot tests shown in Figure 5, the concentration of at least 2.5 mg/mL was enough to inhibit the growth of a spot at ~10^5^ CFU corresponding to samples at ~10^7^ CFU/mL. The natural extract inhibited, in a significant manner, the adherent biomass formation of both strains. In PAO1, in the presence of Cm4-p, the amount of adherent biofilms was ~75% lower than that of the controls (*p* = 0.04), and in B13, the amount of adherent biofilms was ~94% lower (*p* = 0.025), respectively. On the other hand, in both strains, the planktonic biomasses were significantly higher than those in the untreated samples. Indeed, the extract greatly compromised the ability to form biofilms, especially in the hyperproducer strain B13. Upon Cm4-p treatment, the BFI of B13 decreased by ~90-fold, while that of PAO1 decreased by ~4-fold with respect to the untreated counterparts. This could be the result of a direct bactericidal effect of Cm4-p and/or a Quorum Sensing quenching. The time-killing assays showed the bactericidal activity of Cm4-p on *P. aeruginosa* PAO1: a three Log-unit reduction in viable cells was obtained after 6 h (Figure 7). Thus, the impaired biofilm formation could mainly rely on the observed killing effect of the microbial inoculum. Furthermore, in *P. aeruginosa*, environmental and physiological signals tune the complex transcriptional machinery towards adhesion functions and biofilm formation [28]. Under the chosen conditions, the plant extract could subtract cells from this “social” commitment, preventing biofilm formation. As a result, the observed lifestyle change could be due to the ability of Cm4-p to shift microbial populations towards a suspended form rather than an adherent one. Notably, the activity of Cm4-p in inhibiting the biofilm formation of *P. aeruginosa* could be relevant for clinical issues such as the colonization of catheters and prosthesis. As Quorum Sensing (QS) systems are the most relevant actors involved in biofilm formation [29,30], they are undoubtedly antimicrobial promising targets. As mentioned before, Cm4-p extract has a high content of catechin compounds that have been recently proposed as putative QS inhibitors (QSIs). In particular, molecular docking simulations showed the interaction of catechin with LasR, the hierarchically prominent QS regulator in *P. aeruginosa* [31]. The anti-QS and anti-biofilm activities of epigallocatechin-3-gallate (EGCG) were reported by Suqi Hao [32], and Abdel Bar observed the synergistic effect of catechins combined with gallic acid in *P. aeruginosa* [33]. On the basis of these studies, we compared the effect of catechins alone with that of catechins combined with sugar moieties in Cm4-p on biofilm formation.

The administration of 3 g/L of catechin had a pro-biofilm effect, especially in the PAO1 strain, where the adherent phase was 6-fold higher than that of the untreated control, while in the B13 strain, a 1.5-fold increase was observed (Figure 6A). As the effect of pure catechin on the planktonic population was not relevant, we evaluated the anti-biofilm activity of a combination of catechins with glyosidic moieties contained in Cm4-p. This was in agreement with Abdel Bart, who observed good activity upon combining catechin with gallic acid rather than catechin alone [33]. Curiously, both gallic acid and sugar moieties contain different hydroxylic substituents.

#### 2.2.2. Effect of Cm4-p on *P. aeruginosa* Biofilm Eradication

**Figure 8:**
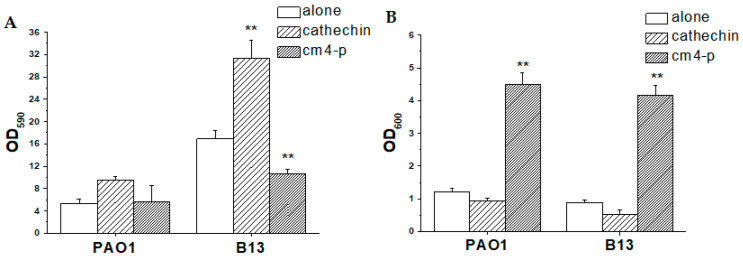
Effects of Cm4-p (3 g/L) and catechin (3 g/L) on *Pseudomonas aeruginosa* PAO1 and B13 biofilm eradication upon 24 h treatment in static conditions. (**A**) Adherent biomass after crystal violet staining (OD_590_) and (**B**) planktonic biomass (OD_600_) upon different treatments. Data are means of three independent experiments ± SD. ** *p* < 0.01.

Biofilms are often responsible for chronic infections of the urinary tract, surgical wounds, venous leg ulcers, diabetic foot ulcers and pressure ulcers. They represent an unlimited reservoir of pathogens [34], and conventional antibiotic and antimicrobial treatments often fail in disrupting well-established biofilms adherent to tissues and clinical devices. As a consequence, it is mandatory to find new strategies that are able to eradicate these microbial communities. Thus, Cm4-p was administered to 24 h old PAO1 and B13 biofilms, and the effect was evaluated 24 h after treatment (Figure 8).

The phytocomplex showed a modest (33%) but statistically significant (*p* = 2.1 × 10^−4^) eradicating effect on the hyperproducer B13 strain compared to the untreated control. This detaching effect was combined with a significant increase in the planktonic counterpart (*p* = 3.1 × 10^−8^), while catechin alone showed significant pro-biofilm activity. On PAO1, Cm4-p shifted the equilibrium versus the planktonic phase without showing any eradicating activity. Thus, the phytocomplex was partially active in eradicating the biofilm hyperproducer B13.

In biofilms, a large number of cells are in a dormant condition and, as a consequence, antibiotics, which are usually effective against dividing bacteria, are not active. In addition, drugs are often adsorbed by the biofilm matrix and prevented from reaching microorganisms [35]. The combination of catechins with the glyosidic components of the phytocomplex could favor the crossing of the sugar matrix formed by Psl, Pel and alginate polysaccharides. As catechins are active in upregulating the expression of glycosyl hydrolases, they could trigger biofilm disassembly by disrupting the exopolysaccharide matrix [36]. Overall, an interesting outcome is that Cm4-p, regardless of the underlying mechanism/s, favors the passing of bacteria from the adherent phase to the suspended one in both strains. In the view of next-generation antimicrobials, the combination of Cm4-p with antibiotics could be a promising chance, as antibiotics could display their activities on bacteria released from the matrix. 

**Figure 9:**
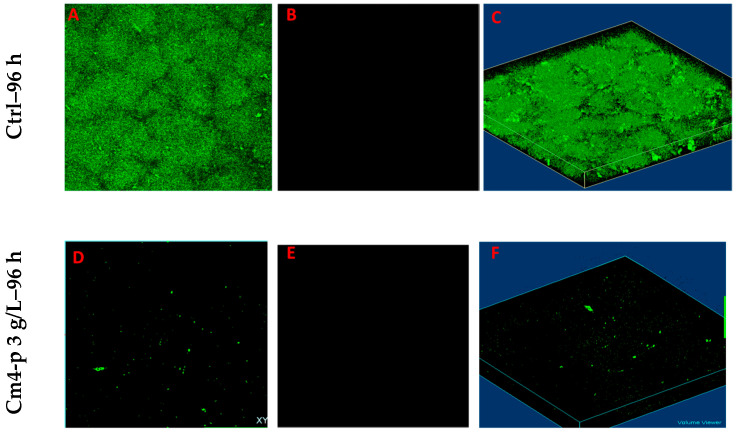
Eradication of formed biofilms under flow conditions (1 mL/min). CLSM images of 96 h old biofilms of *P. aeruginosa* PAO1 grown for 72 h under flow and subjected or not subjected to Cm4-p (12.5 g/L) treatment for 24 h. Green stained cells with intact membrane have to be considered alive (**A**,**D**), whereas red cells with compromised membranes have to be considered dead bacteria (**B**,**E**). Three-dimensional reconstructions of merged channels (live and dead cells) are on right side (**C**,**F**).

The administration of Cm4-p (3 g/L) in flow conditions for 24 h on a 72 h old PAO1 biofilm did not cause any eradication. Thus, the extract concentration was raised to the solubility limit of 12.5 g/L, and as can be observed in Figure 9D–F, this new condition resulted in clear anti-biofilm activity. Notably, the almost complete absence of a red signal (Figure 9E,F) suggests that the treatment with Cm4-p caused a complete eradication of biofilms. In this experimental set-up, we could not evaluate the viability of detached cells. If viable cells were still present, Cmp-4 could be all the same a valuable tool to combine with traditional antibiotics.

## 3. Materials and Methods

### 3.1. Generals

Analytical pure solvents, cyclohexane (CHE), ethanol (EtOH), methanol (MeOH), acetonitrile (MeCN) and bis-(trimethylsilyl)trifluoroacetamide + tertbutyldimethylchlorosilane (MSTFA + 1% TMCS > 95%) were purchased from Sigma-Aldrich (Burlington, MA, USA) and used as received.

### 3.2. Cm4-p Extraction

Cm4-p extract was obtained by modifying the previously reported procedure [22]. Dried leaves of *C. micranthum* (100 g) were manually minced, poured in a 2.5 L brown glass bottle and treated for 24 h at room temperature (RT) under gentle mechanical stirring with 600 mL of CHE. The vegetal material was then separated from the solvent by filtration on a large Buckner funnel, and the leaves were treated, as above, with 95% EtOH. The ethanolic fraction was collected by filtration and evaporated to dryness, providing a solid compound. From this raw material, the purification procedures previously described were carried out, affording Cm4-p. With this extract, water solutions at known weight concentrations were prepared and then used for antimicrobial activity assays and analytical investigations.

### 3.3. Cm4-p Analyses

The Cm4-p extract was analyzed by UV-Vis spectroscopy with a Perkin-Elmer Lambda 10 instrument (PerkinElmer, Waltham, MA, USA), preparing a 1 × 10^−4^ g/L solution in MeOH (wavelength region from 270 to 500 nm, with a step of 1 nm). The FT-IR analysis was carried out with the Nicolet AVATAR FT-IR 360 (Spectralab Scientific Inc., Markham, ON, Canada) by dispersing the sample in KBr disks. Finally, the extract dissolved in D_2_O was analyzed for NMR analysis (recovered on a Bruker 400 MHz spectrometer, Bruker, Billerica, MA, USA) for 1H, 13C and the heterocorrelation.

### 3.4. Gas Chromatography/Mass Spectrometry

Gas chromatographic (GC) separations were performed on a CP-SIL 5 capillary column (30 m length × 0.25 mm i.d. and 1 µm film thickness; Varian, Leini, Italy) using FOCUS gas chromatography equipped with a DSQ II mass spectrometer (Thermo Scientific, Rodano, Italy) with an electron impact (EI) source operating at 70 eV.

An aliquot of 5 mg of Cm4-p was dissolved in 10 mL of MeOH; from this mother solution, a water dilution of 1:50 (total volume 10 mL) gave a sample concentration of 1.0 × 10^−2^ g/L (10 ppm). Two sequential 1:10 dilutions were further made to prepare Cm4-p sample at concentrations of 1 ppm and 0.1 ppm, respectively. An aliquot of 100 μL of each diluted solution was dried under gentle N_2_ stream, and silylation of hydroxyl groups was carried out by treating samples with mixtures composed of 40 μL MeCN and 60 μL of MSTFA + TMCS 1%. Derivatization processes were performed in sealed vials at 37 °C for 4 h. After this period, 1 μL of silylated Cm4-p was analyzed by means of GC/MS according to the conditions reported below.

GC/MS analyses were performed under these conditions: the oven temperature was kept at 150 °C for 1 min and then raised to 190 °C at 15 °C/min, held for 6 min and then raised to 280 °C and maintained at this value for 20 min. Injector was set at 250 °C, transfer line was set at 250 °C and carrier gas (He) was set at 1.0 mL/min constant flow. Mass instrument ion source was set at 300 °C, the acquisition started 5 min after injection, scanned masses ranged from 50 to 700 *m*/*z* with a scan speed of 1 scan/s. The components were identified through recognition with the NIST library.

### 3.5. Bacterial Strains

The Cm4-p extract was tested against *P. aeruginosa* PAO1 [37] and its isogenic derivative B13, a biofilm hyperproducer transposon mutant that was previously isolated [25].

### 3.6. Time-Killing Assay

Time-killing studies were performed according to the CLSI guidelines (formerly National Committee for Clinical Laboratory Standards, Approved Standards M7-A4, Wayne, 1997), which were modified as necessary for the extract under study. *P. aeruginosa* cells were grown overnight in LB medium at 37 °C with agitation, and then the culture was diluted to a final concentration of 10^5^ CFU/mL and incubated with Cm4-p (3 g/L) at 37 °C under shaking at 150 rpm for 36 h. Samples were collected at periods of 0, 30, 80 and 160 min and 6 h and 36 h after treatment. At each time point, bacterial concentration was determined by viable count technique and expressed as CFU/mL; to this aim, a volume (0.01 mL) of undiluted or serially diluted samples was plated on LB agar and incubated for 24 h at 37 °C.

### 3.7. Spot Test

An overnight culture of P. aeruginosa grown in LB medium at 37 °C with agitation was centrifuged at 4000× *g* for 10 min, washed twice with one volume of 1× phosphate buffer (KH_2_PO_4_/K_2_HPO_4_, 10 mM, pH 7.4) and suspended in one volume of the same solution. Volumes of 200 µL (of 1× phosphate buffer) deriving from the original cell suspension or from ten-fold serial dilutions were added to a 96 multi-well plate and incubated alone or in the presence of decreasing concentrations of the extract. After 6 h of incubation at room temperature, bacteria were inoculated on LB agar. After incubation at 37 °C for 24 h, growth spots were analyzed. Experiments were performed in triplicate.

### 3.8. Evaluation of Anti-Biofilm Activity of Cm4-p on P. aeruginosa Strains 

Wild-type *P. aeruginosa* PAO1 and B13 mutant strains were grown overnight in M9 medium with the addition of 10 mM of glucose and were 100-fold diluted in fresh medium to inoculate a 12 well plate. To evaluate the inhibition of biofilm formation, Cm4-p or pure catechin (Sigma Aldrich) was added to cells at a final concentration of 3 g/L. After 24 h of incubation at 37 °C, planktonic biomass was removed, and OD (optical density) at 600 nm was measured. Biofilms were stained with Crystal Violet (CV) 0.1% for 15 min, washed twice with phosphate buffer and dried for 2 h. The amount of CV attached to biofilms was measured by treating samples with acetic acid 30% for 10 min and measuring the amount of solubilized dye spectrophotometrically at 590 nm. The BFI (Biofilm Forming Index) was determined by applying the formula BFI = (AB − CW)/G, in which AB is the OD of the stained attached microorganisms, CW is the optical density of the stained control wells containing only medium and G is the optical density of the cells grown in suspended cultures [38].

To evaluate the efficacy in biofilm eradication, Cm4-p or catechin was added, at a concentration of 3 g/L, to 24 h old biofilms which were allowed to grow for a further 24 h at 37 °C. CV staining was performed as described above.

### 3.9. Evaluation of Anti-Biofilm Activity of Cm4-p on Biofilms Grown under Flow 

**Figure 10:**
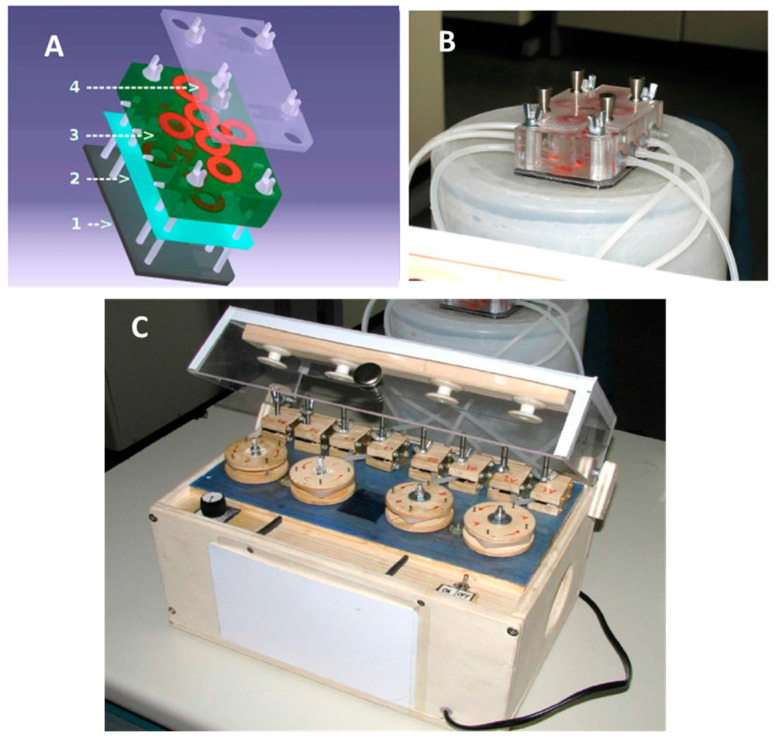
A biofilm chamber box (mounted assembly). The 3D project (**A**) and built model (**B**): (1) support plate, (2) biofilm support material, (3) chamber basement, (4) chamber cover (without injectors). The four chambers in the central line are designed for dynamic biofilm growth while the two in the middle, above and below the central chamber line are designed for static biofilm incubation. A peristaltic pump (**C**) was connected with four rotors that rotate at the same speed in order to flow the medium at 1 mL/min.

The prototype of the flow chamber box built for this study is presented in Figure 10.

The flow chamber box is a modular system (Figure 10A,B) composed of three parts. On the bottom, a metallic plate constitutes the surface on which a biofilm support of various materials (glass, plastic) is placed. In the middle of the chamber, there is a block with six wells (four designed for growth under flow and two for static incubation) and plastic input/output tube connectors. A top cover is placed for sterility purposes and to prevent evaporation. The input (inlet) connector is positioned near the top of each round well, while the output connector (drain) is placed near the bottom. Pin injectors with a diameter of 1 mm are placed on the cover surface and sealed with external rubber disks. This chamber was sterilized by a treatment with a 50% ethanol aqueous solution for 10 min, followed by drying under a vacuum (rotary vacuum pump) for 24 h. A peristaltic pump (Figure 10C) was connected to the flow chamber box to create a tunable flux of 1 mL/min.

To evaluate the eradication effect of Cm4-p on the formed biofilms, *P. aeruginosa* PAO1 was grown overnight in LB (Lysogeny Broth) medium and diluted to 10^5^ CFU/mL in M9 medium supplemented with 10 mM glucose. A glass disk was placed in each of the four-chamber bases for biofilm adhesion. Each of the four growth chambers was filled with 4 mL of the bacterial culture. The chambers were pre-incubated in the absence of flow for 1 h, and the flow was applied for the following 72 h at 37 °C. After the addition of Cm4-p (3 g/L or 12.5 g/L) or distilled water, incubation was further performed for 24 h at 37 °C under flow. Biofilm chambers were then recovered after the removal of the planktonic phases, unlinking each inlet tube from its respective chamber. BacLight^®^ kit (Invitrogen, Carlsbad, CA, USA) reagents (20 μL) were added to each chamber, and after 15 min, each stained disk was laid onto a cover glass for further Confocal Laser Scanning Microscopy (CLSM) analyses. CLSM acquisitions were performed with a Leica TCS SP5 (Leica Microsystems, Wetzlar, Germany). Biofilms’ 3D models were obtained using V3D 2.801 under Linux environment [39].

### 3.10. Statistical Analyses

Statistical analyses of the experimental data (at least 3 independent tests) were performed using one-way ANOVA followed by Duncan’s post hoc test. The ANOVA test was performed by considering data with a *p*-value less than 0.05 as significant. Data normality was tested using the Shapiro–Wilk test [40].

## 4. Conclusions

The extract from dried leaves of *C. micranthum* G. Don, named Cm4-p, is a phytocomplex characterized by the presence of catechin and sugar moieties. This extract seems to be a very promising compound as it inhibited the biofilm formation of *P. aeruginosa* PAO1 at a concentration of 3 g/L. At a higher concentration (12.5 g/L), it was able to eradicate 72 h old PAO1 biofilms under flow conditions. Interestingly, its activity was also observed against the hyperproducer biofilm B13 strain, a PAO1 derivative known to enrich the exopolysaccharide part of the biofilm.

As pure catechin alone showed pro-biofilm activity, the glycosidic components seem to be essential to confer anti-pseudomonal activity to the phytocomplex. Further investigations will be focused on determining the glycosidic skeleton of the phytocomplex.

## Data Availability

Data are contained within the article.

## References

[1] Gellatly S.L., Hancock R.E. (2013). Pseudomonas aeruginosa: New insights into pathogenesis and host defenses. Pathog. Dis..

[2] Lambert P.A. (2002). Mechanisms of antibiotic resistance in Pseudomonas aeruginosa. J. R. Soc. Med..

[3] Stewart P.S., Costerton J.W. (2001). Antibiotic resistance of bacteria in biofilms. Lancet.

[4] Hall-Stoodley L., Stoodley P. (2009). Evolving concepts in biofilm infections. Cell. Microbiol..

[5] Hoiby N., Ciofu O., Johansen H.K., Song Z.J., Moser C., Jensen P.O., Molin S., Givskov M., Tolker-Nielsen T., Bjarnsholt T. (2011). The clinical impact of bacterial biofilms. Int. J. Oral Sci..

[6] Anderson G.G., O’Toole G.A. (2008). Innate and induced resistance mechanisms of bacterial biofilms. Curr. Top. Microbiol. Immunol..

[7] Desrousseaux C., Sautou V., Descamps S., Traore O. (2013). Modification of the surfaces of medical devices to prevent microbial adhesion and biofilm formation. J. Hosp. Infect..

[8] Banin E., Brady K.M., Greenberg E.P. (2006). Chelator-induced dispersal and killing of cells in a biofilm. Appl. Environ. Microbiol..

[9] Chen X., Stewart P.S. (2000). Biofilm removal caused by chemical treatments. Water Res..

[10] Kolodkin-Gal I., Romero D., Cao S., Clardy J., Kolter R., Losick R. (2010). D-amino acids trigger biofilm disassembly. Science.

[11] Orlandi V.T., Rybtke M., Caruso E., Banfi S., Tolker-Nielsen T., Barbieri P. (2014). Antimicrobial and anti-biofilm effect of a novel BODIPY photosensitizer against Pseudomonas aeruginosa PAO1. Biofouling.

[12] Hostettmann K., Marston A. (2002). Twenty years of research into medicinal plants: Results and perspectives. Phytochem. Rev..

[13] Calvo M., Arosemena E., Shiva C., Adelantado C. (2012). Antimicrobial activity of plant natural extracts and essential oils. Science against Microbial Pathogens: Communicating Current Research and Technological Advances.

[14] Saleem M., Nazir M., Ali M.S., Hussain H., Lee Y.S., Riaz N., Jabbar A. (2010). Antimicrobial natural products: An update on future antibiotic drug candidates. Nat. Prod. Rep..

[15] Tan F., Shi S., Zhong Y., Gong X., Wang Y. (2002). Phylogenetic relationships of Combretoideae (Combretaceae) inferred from plastid, nuclear gene and spacer sequences. J. Plant Res..

[16] Watt J.M. (1962). The Medicinal and Poisonous Plants of Southern and Eastern Africa; Being an Account of Their Medicinal and Other Uses, Chemical Composition, Pharmacological Effects and Toxicology in Man and Animal.

[17] Van Wyk B. (2013). Field Guide to Trees of Southern Africa.

[18] Masoko P., Picard J., Eloff J.N. (2005). Antifungal activities of six South African Terminalia species (Combretaceae). J. Ethnopharmacol..

[19] McGaw L.J., Rabe T., Sparg S.G., Jager A.K., Eloff J.N., van Staden J. (2001). An investigation on the biological activity of Combretum species. J. Ethnopharmacol..

[20] Martini N., Eloff J.N. (1998). The preliminary isolation of several antibacterial compounds from Combretum erythrophyllum (Combretaceae). J. Ethnopharmacol..

[21] Katerere D.R., Gray A.I., Nash R.J., Waigh R.D. (2003). Antimicrobial activity of pentacyclic triterpenes isolated from African Combretaceae. Phytochemistry.

[22] Banfi S., Caruso E., Orlandi V., Barbieri P., Cavallari S., Viganò P., Clerici P., Chiodaroli L. (2014). Antibacterial activity of leaf extracts from Combretum micranthum and Guiera senegalensis (Combretaceae). Res. J. Microbiol..

[23] Morita Y., Tomida J., Kawamura Y. (2014). Responses of Pseudomonas aeruginosa to antimicrobials. Front. Microbiol..

[24] Wei Q., Ma L.Z. (2013). Biofilm matrix and its regulation in Pseudomonas aeruginosa. Int. J. Mol. Sci..

[25] Orlandi V.T., Bolognese F., Martegani E., Cantaluppi V., Medana C., Barbieri P. (2017). Response to photo-oxidative stress of Pseudomonas aeruginosa PAO1 mutants impaired in different functions. Microbiology.

[26] Moscoso J.A., Mikkelsen H., Heeb S., Williams P., Filloux A. (2011). The Pseudomonas aeruginosa sensor RetS switches type III and type VI secretion via c-di-GMP signalling. Environ. Microbiol..

[27] Bhagirath A.Y., Pydi S.P., Li Y., Lin C., Kong W., Chelikani P., Duan K. (2017). Characterization of the Direct Interaction between Hybrid Sensor Kinases PA1611 and RetS That Controls Biofilm Formation and the Type III Secretion System in Pseudomonas aeruginosa. ACS Infect. Dis..

[28] Vohra M., Kour A., Kalia N.P., Kumar M., Sharma S., Jaglan S., Kamath N., Sharma S. (2024). A comprehensive review of genomics, transcriptomics, proteomics, and metabolomic insights into the differentiation of Pseudomonas aeruginosa from the planktonic to biofilm state: A multi-omics approach. Int. J. Biol. Macromol..

[29] Vadakkan K., Ngangbam A.K., Sathishkumar K., Rumjit N.P., Cheruvathur M.K. (2024). A review of chemical signaling pathways in the quorum sensing circuit of Pseudomonas aeruginosa. Int. J. Biol. Macromol..

[30] Lee J., Zhang L. (2015). The hierarchy quorum sensing network in Pseudomonas aeruginosa. Protein Cell.

[31] Chaieb K., Kouidhi B., Hosawi S.B., Baothman O.A.S., Zamzami M.A., Altayeb H.N. (2022). Computational screening of natural compounds as putative quorum sensing inhibitors targeting drug resistance bacteria: Molecular docking and molecular dynamics simulations. Comput. Biol. Med..

[32] Hao S., Yang D., Zhao L., Shi F., Ye G., Fu H., Lin J., Guo H., He R., Li J. (2021). EGCG-Mediated Potential Inhibition of Biofilm Development and Quorum Sensing in Pseudomonas aeruginosa. Int. J. Mol. Sci..

[33] Abdel Bar F.M., Alossaimi M.A., Elekhnawy E., Alzeer M.A.A., Abo Kamer A., Moglad E., ElNaggar M.H. (2022). Anti-Quorum Sensing and Anti-Biofilm Activity of Pelargonium x hortorum Root Extract against Pseudomonas aeruginosa: Combinatorial Effect of Catechin and Gallic Acid. Molecules.

[34] Su Y., Yrastorza J.T., Matis M., Cusick J., Zhao S., Wang G., Xie J. (2022). Biofilms: Formation, Research Models, Potential Targets, and Methods for Prevention and Treatment. Adv. Sci..

[35] Yin R., Cheng J., Wang J., Li P., Lin J. (2022). Treatment of Pseudomonas aeruginosa infectious biofilms: Challenges and strategies. Front. Microbiol..

[36] Yu S., Su T., Wu H., Liu S., Wang D., Zhao T., Jin Z., Du W., Zhu M.J., Chua S.L. (2015). PslG, a self-produced glycosyl hydrolase, triggers biofilm disassembly by disrupting exopolysaccharide matrix. Cell Res..

[37] Stover C.K., Pham X.Q., Erwin A.L., Mizoguchi S.D., Warrener P., Hickey M.J., Brinkman F.S., Hufnagle W.O., Kowalik D.J., Lagrou M. (2000). Complete genome sequence of Pseudomonas aeruginosa PAO1, an opportunistic pathogen. Nature.

[38] Teh K.H., Flint S., French N. (2010). Biofilm formation by Campylobacter jejuni in controlled mixed-microbial populations. Int. J. Food Microbiol..

[39] Peng H., Ruan Z., Long F., Simpson J.H., Myers E.W. (2010). V3D enables real-time 3D visualization and quantitative analysis of large-scale biological image data sets. Nat. Biotechnol..

[40] Tabachnick B.G., Fidell L.S. (2019). Using Multivariate Statistics.

